# Beyond Vascular Territories: The Dual Pathology of Posterior Cerebral Artery Stroke With Reversible Cytotoxic Splenial Lesion

**DOI:** 10.7759/cureus.119479

**Published:** 2026-11-11

**Authors:** Bethan Laura Whitwell, Nasirodiin Haroun, Dawood Sohail

**Affiliations:** 1 Renal Medicine, Queen Elizabeth Hospital Birmingham, Birmingham, GBR; 2 Cardiology, Heartlands Hospital, Birmingham, GBR; 3 Stroke Medicine, Queen Elizabeth Hospital Birmingham, Birmingham, GBR

**Keywords:** acute ischaemic stroke (ais), clocc, dual pathology, stroke imaging, stroke management

## Abstract

The authors present a case highlighting an important stroke mimic: a cytotoxic lesion of the corpus callosum (CLOCC).

This case report describes a 28-year-old male with pre-existing hypertension, poorly controlled type 2 diabetes, high BMI, and multiple atherosclerotic risk factors, including polysubstance use (cannabis, cigarettes, vaping), who presented with acute-onset left-sided weakness, blurred vision, and paraesthesia. Initial non-contrast CT head was unremarkable. Subsequent MRI demonstrated acute signal changes involving the splenium of the corpus callosum and the tail of the right hippocampus. CT angiography revealed an occlusion of the right posterior cerebral artery at the P2 segment; however, the atypical location for conventional ischaemia raised suspicion for an alternative or concurrent cytotoxic aetiology. Given the patient's markedly poor glycaemic control, a metabolic trigger was strongly considered. Serial MRI imaging was performed, and further supported this hypothesis by demonstrating near-complete regression of the abnormal signal within the splenium following glycaemic optimisation, whilst also correlating with clinical improvement. Management comprised dual anti-platelet therapy, strict glucose and blood pressure control, and lifestyle modification.

This case underscores the importance of considering metabolic and cytotoxic aetiologies alongside conventional ischaemia in young stroke presentations, particularly where imaging findings are atypical for vascular territory. It highlights the diagnostic complexity of acute neurological presentations in young patients with significant vascular and metabolic risk factors, demonstrating how severe hyperglycaemia can mimic or coexist with acute ischaemic stroke. Atypical MRI findings for a PCA occlusion seen in this case emphasise the importance of considering reversible cytotoxic lesions, such as CLOCCs, as a differential diagnosis.

## Introduction

The authors present this case to increase awareness of an important potential stroke mimic--a cytotoxic lesion of the corpus callosum (CLOCC)--in order to promote early diagnosis and therefore improved clinical outcomes. CLOCCs describe reversible secondary lesions appearing as restricted diffusion on diffusion-weighted imaging (DWI) [1] and can often closely mimic ischaemic stroke in presentation.

This case report describes a 28-year-old male with pre-existing hypertension, poorly controlled type 2 diabetes, high body mass index (BMI), and polysubstance use (cannabis, cigarettes, vaping) who presented with acute-onset left-sided weakness, blurred vision, and paraesthesia. Despite initial suspicion of an ischaemic stroke, and computed tomography angiography (CTA) demonstrating an occlusion of the right posterior cerebral artery (PCA), the atypical location for conventional ischaemia raised suspicion of an alternative or concurrent cytotoxic aetiology. Given the patient’s markedly poor glycaemic control, a metabolic trigger was strongly considered. Serial magnetic resonance imaging (MRI) demonstrated near-complete regression of the abnormal signal within the splenium following glycaemic optimisation, thus further supporting this potential hypothesis. Management comprised dual antiplatelet therapy, strict glucose and blood pressure control, and lifestyle modification. This case highlights the importance of the consideration of metabolic and cytotoxic aetiologies alongside conventional ischaemia, particularly where imaging findings are atypical for vascular territory.

Ischaemic strokes, defined as strokes caused by vascular occlusion or stenosis, account for approximately 87% of all strokes [2]. Age has been widely acknowledged as a risk factor for ischaemic stroke; however, up to 15% of strokes now occur in those under 55 [3], with an increase in incidence of up to 90% demonstrated in Oxfordshire, United Kingdom, between 1981-1986 and 2010-2018 [3]. Although the exact cause for this is unknown, possibilities include increasing prevalence of traditional modifiable risk factors (e.g., high BMI, smoking, hypertension, diabetes), air pollution, long working hours, psychosocial stress, and illicit drug use [3]. Classical stroke presentation may include motor and/or sensory deficits, altered consciousness, speech disturbance, visual disturbance, loss of balance, and dizziness. Early recognition and management are vital, as tissue-saving treatment with thrombolysis and thrombectomy is time-critical; however, accurate diagnosis of stroke can be challenging, with some studies suggesting stroke mimics may account for approximately 25% of all possible stroke presentations [4]. These include peripheral vestibular dysfunction, toxic/metabolic causes (e.g., CLOCCs), seizures, functional disorders, and migraines.

CLOCCs are cytotoxic lesions observed in the corpus callosum. They may be associated with drugs, malignancy, infection, subarachnoid haemorrhage, metabolic disorders, and trauma [1]. The pathophysiology of CLOCCs is believed to be associated with an inflammatory cascade, with markedly increased levels of cytokines and extracellular glutamate via cell-cytokine interactions, ultimately leading to callosal neuron and microglia dysfunction. This, in turn, leads to cytotoxic oedema as water is trapped within the malfunctioning cells [1]. The splenium of the corpus callosum is particularly affected due to its high density of glutamate receptors and oligodendrocytes [5]. Diagnosis involves traditional stroke investigations, including routine blood tests, electrocardiogram (ECG), computed tomography (CT) head, CTA, and MRI head. CLOCCs typically appear as non-enhancing, hyperintense areas on MRI, with restricted diffusion, and often near-complete resolution over time [5]. Differentiating between CLOCC and ischaemia on imaging can be challenging, and misdiagnosis is common. Management of CLOCCs largely revolves around the underlying cause of the lesion. This may include optimisation of blood pressure, drug withdrawal, treatment of malignancy, and optimisation of glucose control.

## Case presentation

A 28-year-old male with a background of hypertension and type 2 diabetes was brought in by ambulance with sudden-onset left-sided limb weakness and frontal headache, which self-resolved within one hour. He had a history of cannabis and cigarette smoking, vaping, and occasional alcohol excess. His BMI at the time of presentation was 40.67 kg/m^2^. Initial workup involved an ECG, demonstrating normal sinus rhythm with left ventricular hypertrophy and benign early repolarisation, and blood tests which revealed elevated cholesterol, haemoglobin A1c (HbA1c) and alanine transaminase (ALT), with low high-density lipoprotein (HDL) (Table 1). CT head and transcranial Doppler ultrasound did not detect any acute abnormality. The patient was therefore discharged home with two weeks of aspirin and a referral to the transient ischaemic attack (TIA) clinic. 

**Table 1:** Blood test results on initial presentation

Test	Result	Normal Reference Range	Interpretation
C-Reactive Protein (CRP)	4mg/L	0-5mg/L	Within range
Alanine Transaminase (ALT)	60IU/L	0-55IU/L	Above reference range
Alkaline Phosphatase (ALP)	89U/L	30-130U/L	Within range
Cholesterol	6.6mmol/L	<4.0mmol/L	Above reference range
High-Density Lipoprotein (HDL)	0.87mmol/L	>1.55mmol/L	Below reference range
Low-Density Lipoprotein (LDL)	4.95mmol/L	<3.0mmol/L	Above reference range
Haemoglobin A1c (HBA1c)	109mmol/mol (12.1%)	20-42mmol/mol (4-6%)	Above reference range

Eight days post-discharge, the patient was admitted from clinic with a one-week history of intermittent episodes of left arm weakness and tingling, blurred vision, and headaches lasting for a few minutes. On admission, his National Institutes of Health Stroke Scale (NIHSS) was zero [6], his Glasgow Coma Score (GCS) was 15/15, and he had no neurological signs or symptoms. During this admission, a transthoracic echocardiogram (TTE) was performed, which showed left ventricular hypertrophy. The diabetes team was also consulted in light of his elevated HbA1c and blood glucose results throughout admission (ranging from 7-10 mmol/L pre-meal), who advised an increase in his dose of metformin to 1g twice daily (BD), and to pause dapagliflozin due to the risk of euglycaemic diabetic ketoacidosis. MRI brain was also completed, which reported an acute infarction involving the splenium of the corpus callosum (Figure 1), with an acute infarction also in the tail of the right hippocampus (Figure 2). This is further highlighted on the corresponding apparent diffusion coefficient (ADC) map (Figure 3).

**Figure 1:**
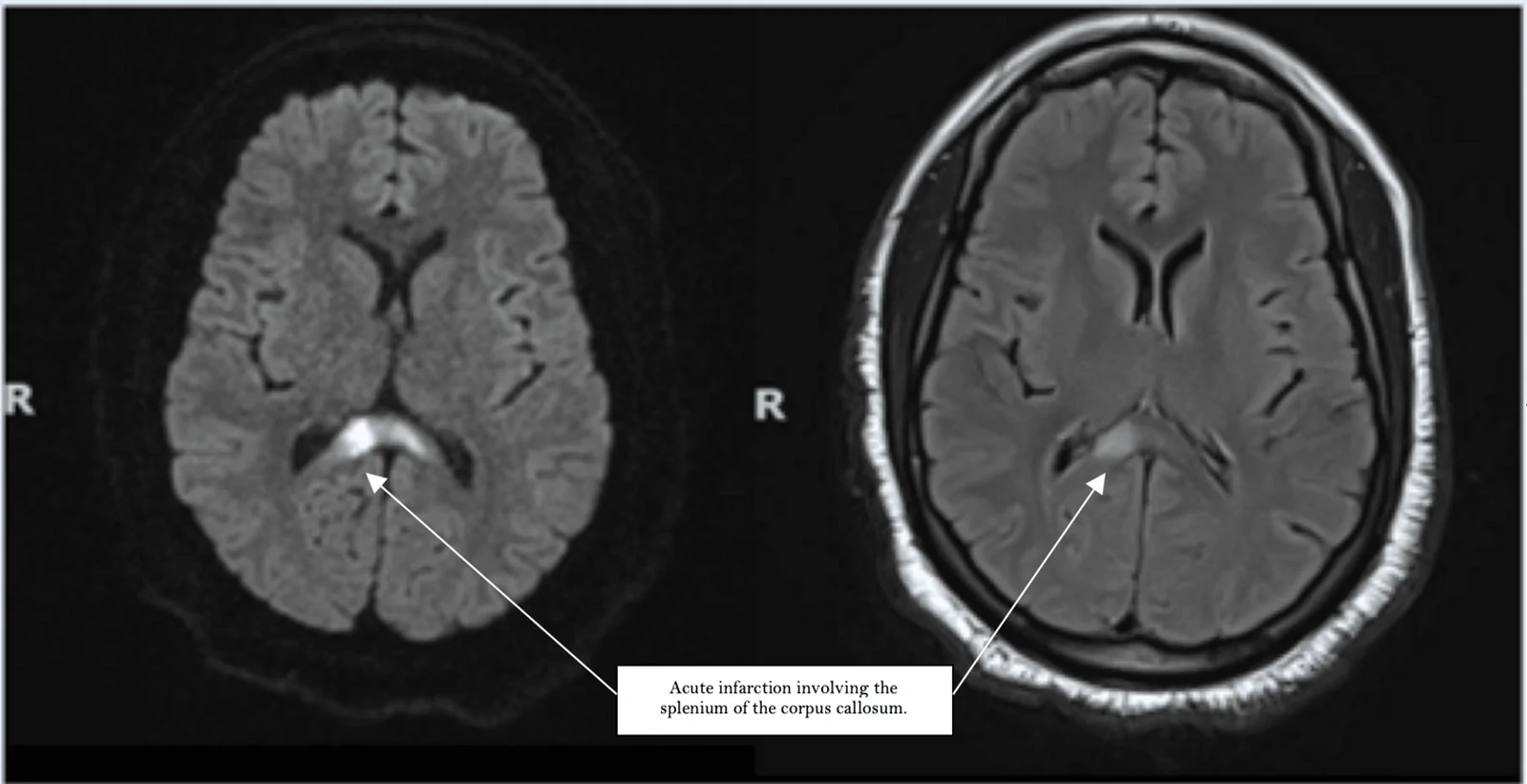
DWI (left) and FLAIR (right) following initial presentation, demonstrating acute infarction involving the splenium of the corpus callosum 'R' indicates the right side on both DWI and FLAIR. DWI: diffusion-weighted imaging; FLAIR: fluid-attenuated inversion recovery

**Figure 2:**
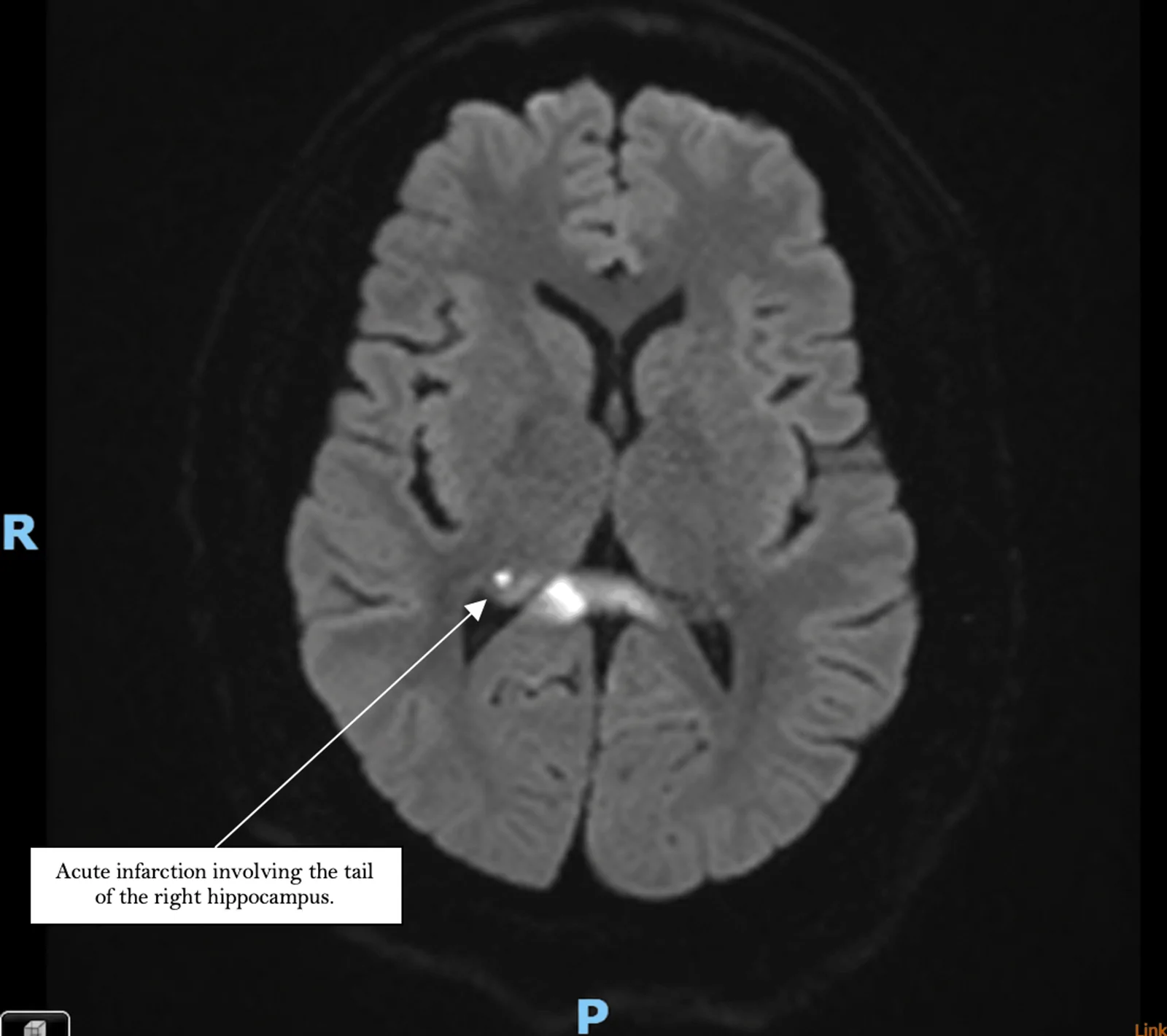
DWI following initial presentation, demonstrating acute infarction involving the tail of the right hippocampus 'R' indicates the right side. DWI: diffusion-weighted imaging

**Figure 3:**
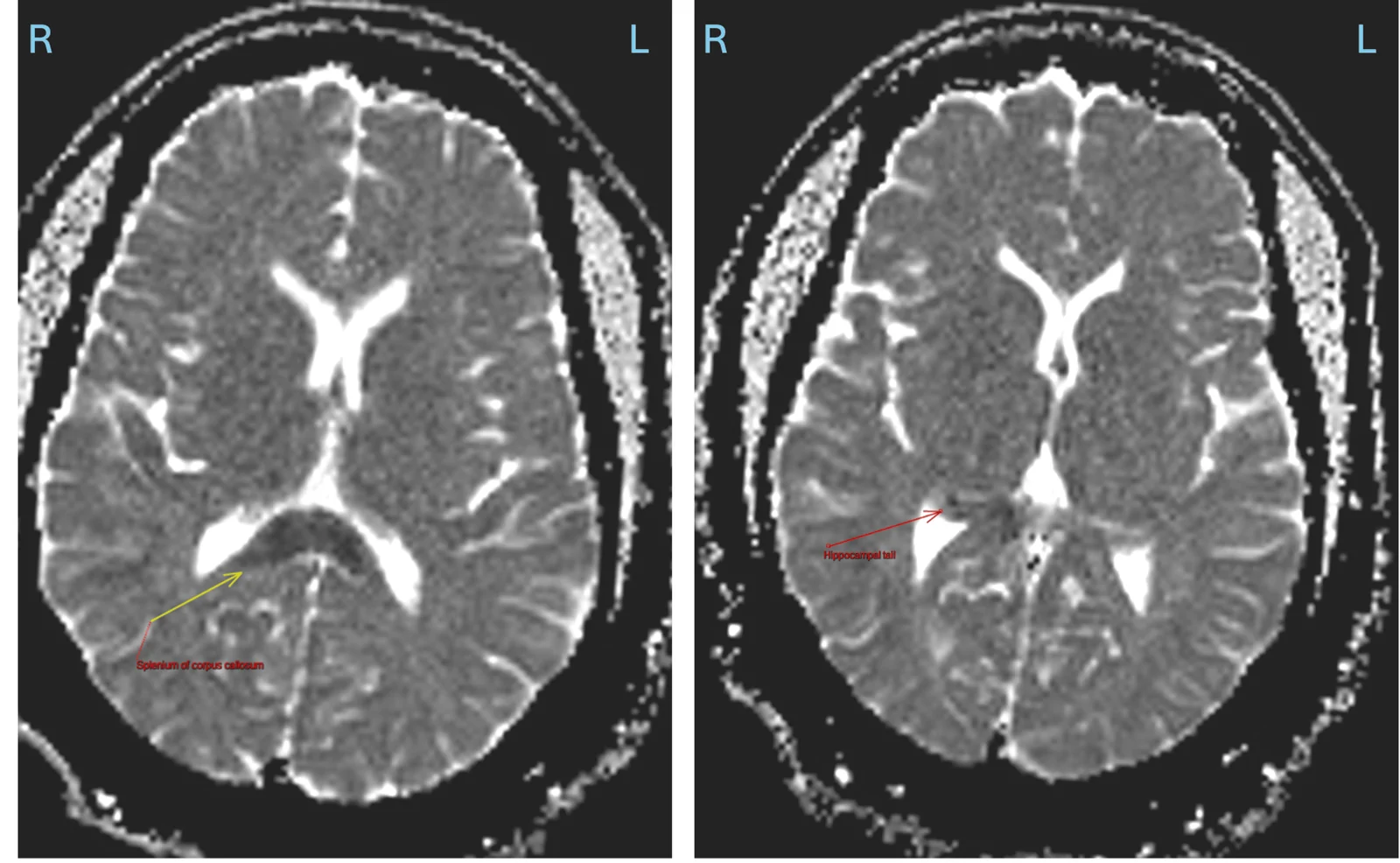
Corresponding ADC map from the initial MRI following presentation demonstrating restricted diffusion in the tail of the right hippocampus, separate from the splenial lesion. The yellow arrow in the left image points to the area of restricted diffusion in the splenium of the corpus callosum, whilst the red arrow in the right image points to the area of restricted diffusion in the tail of the right hippocampus. 'R' indicates the right side on both images. ADC: apparent diffusion coefficient

Consequently, the patient underwent a young stroke workup and was started on dual antiplatelet therapy (DAPT) with clopidogrel and aspirin. Notably, CT angiogram revealed poor/no opacification of the P2 segment and non-opacification of the P3 segment of the right PCA, reflecting an occluded right PCA from the P2 segment (Figure 4). This further supported a diagnosis of an ischaemic stroke. The patient was therefore provided with lifestyle advice with respect to smoking and glucose control, and started DAPT (thrombolysis and thrombectomy were not considered due to the delayed presentation following symptom onset, in accordance with clinical guidelines [7]) (Figure 5). He was also started on losartan for blood pressure control, and a secondary hypertension screen was completed. This included an MRI of the heart, which reported marked left ventricular hypertrophy that was deemed likely hypertensive heart disease. Cardiology review further supported the previously given weight loss advice and adequate blood pressure control. Following full workup and commencement of the appropriate treatment, the patient was discharged from the hospital for repeat MRI head in six weeks.

**Figure 4:**
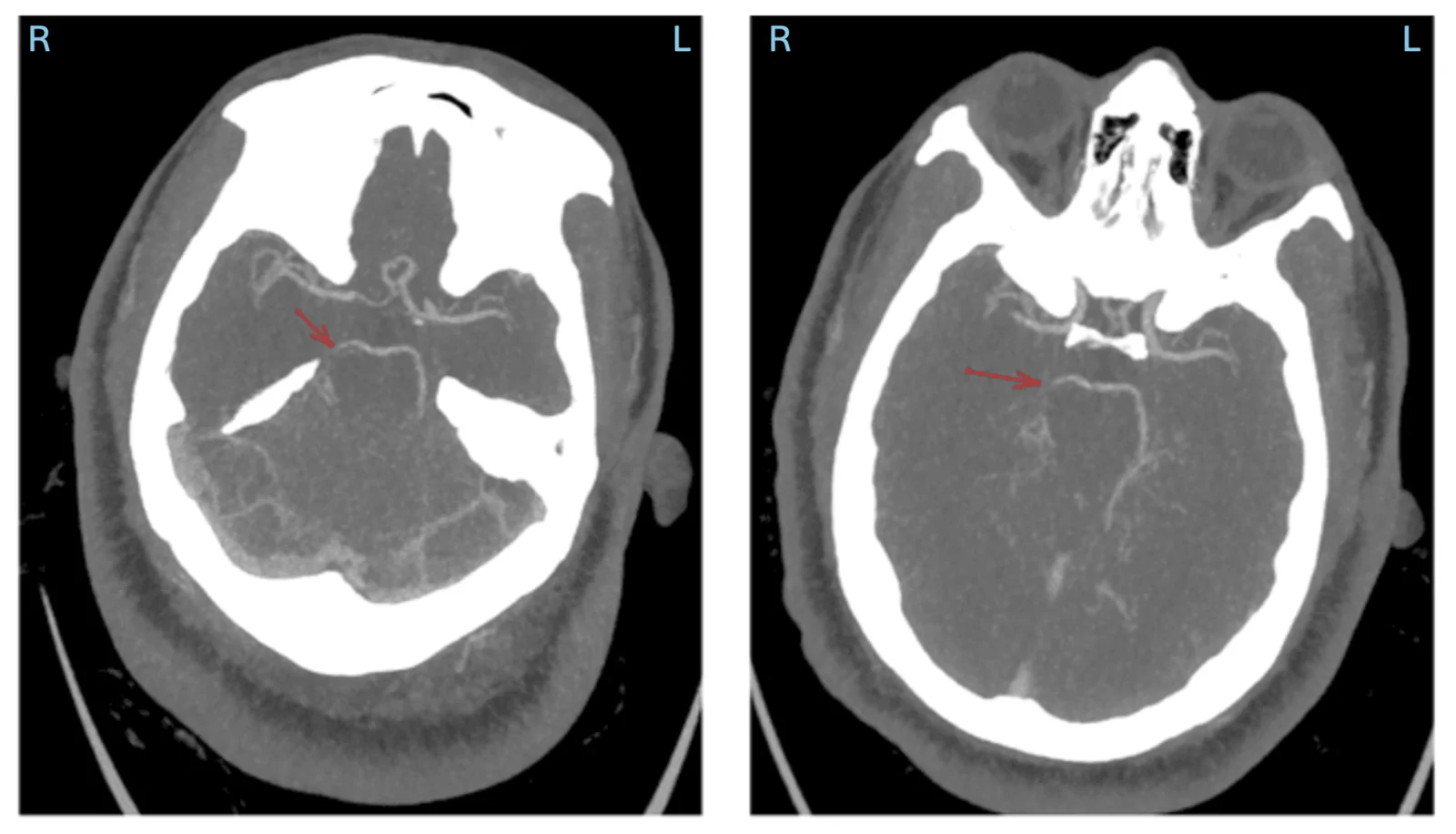
CT angiography demonstrating the posterior cerebral arteries (P1-P3 segments), with occlusion of the right posterior cerebral artery at the P2 segment, indicated by the red arrows on both images. 'R' indicates the right side on both images.

**Figure 5:**
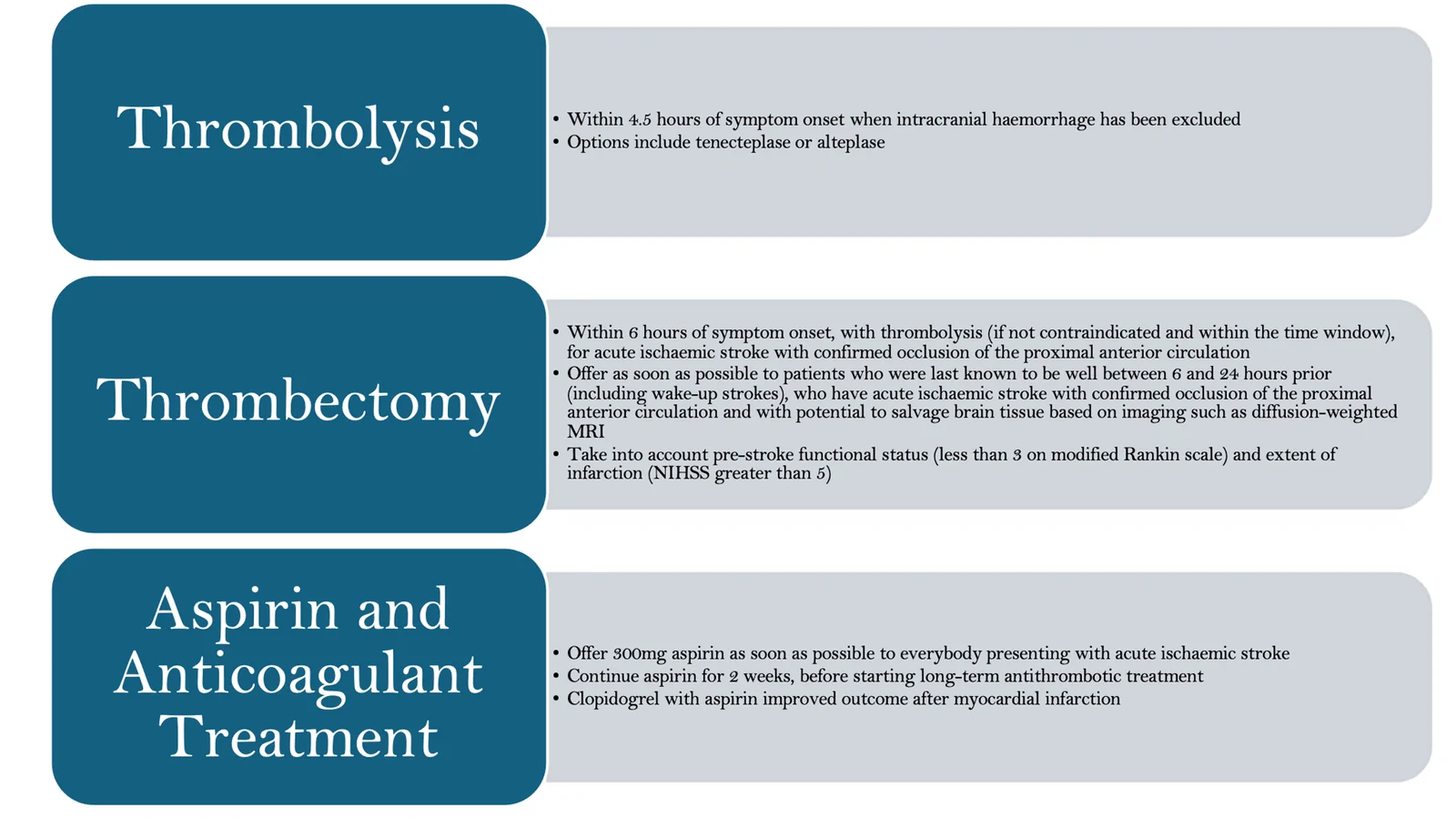
Recommended management options for acute ischaemic stroke Adapted from the NICE guidelines for the management of stroke [7]

Serial MRI head scans were completed following the patient’s discharge. Fourteen weeks after the initial MRI, a repeat MRI head reported residual changes within the splenium of the corpus callosum of the right cerebral hemisphere, which did not show progression or regression between studies. It was also noted on this scan that the lesion was slightly bright on diffusion-weighted sequences and was atypical for an ischaemic lesion and more likely to be demyelinating. This raised suspicion for an alternative or concurrent cytotoxic aetiology. Given the patient’s markedly poor glycaemic control, a metabolic trigger was strongly considered. A subsequent MRI head further supported this possibility by demonstrating near-complete regression of the abnormal T2 high signal within the right side of the splenium of the corpus callosum following glycaemic optimisation (Figure 6). This MRI was taken 18 weeks after the initial MRI (Figure 1) and correlated with an HbA1c of 63 mmol/mol, a dramatic drop from the previously reported 109 mmol/mol on initial presentation.

**Figure 6:**
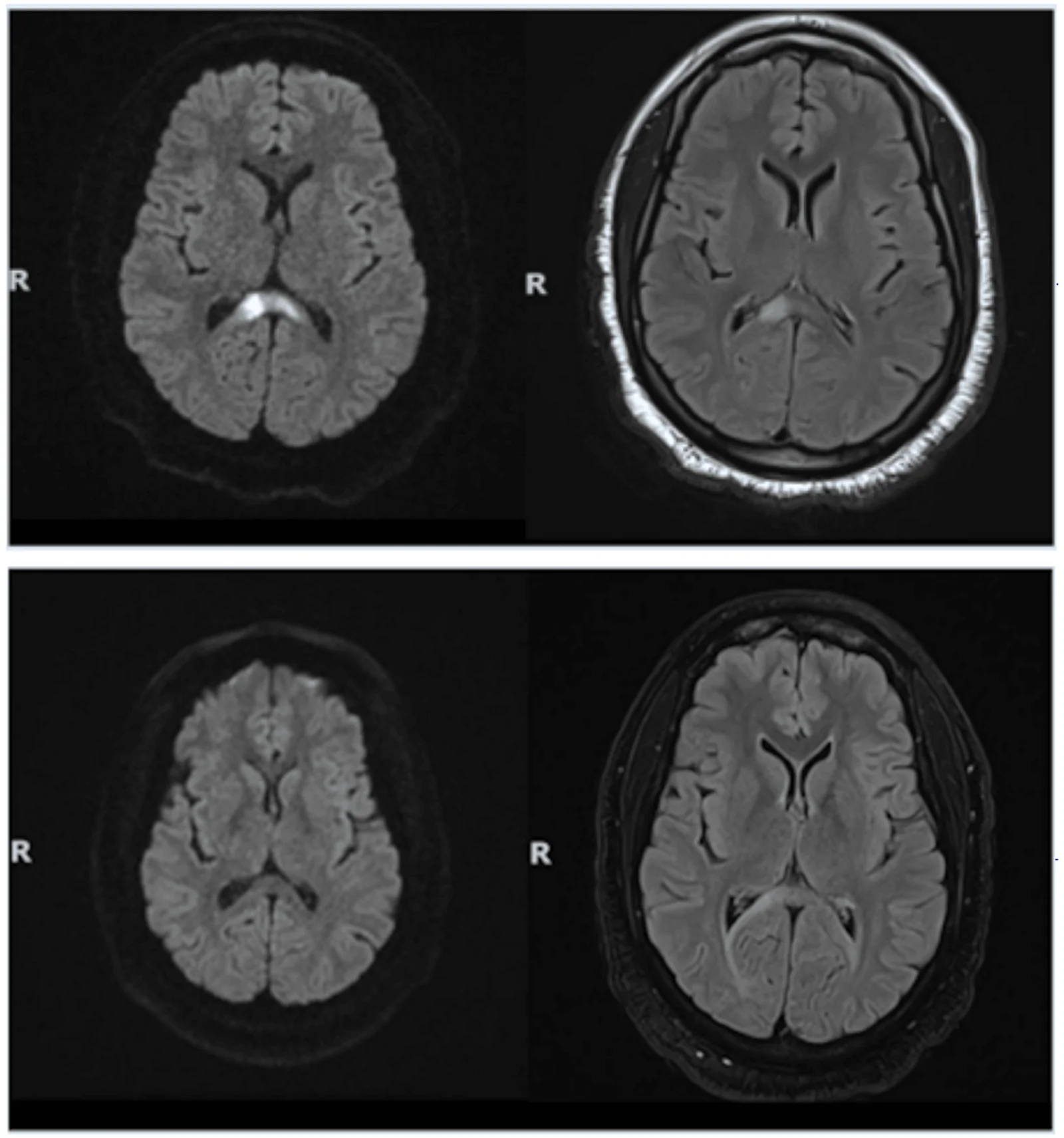
DWI (left) and FLAIR (right) demonstrating near-complete resolution. Images taken on initial presentation are shown directly above to demonstrate the significant changes appreciated on serial imaging. 'R' indicates the right side on both DWI and FLAIR. DWI: diffusion-weighted imaging; FLAIR: fluid-attenuated inversion recovery

## Discussion

Stroke is traditionally associated with age; however, studies have found that 5-15% of strokes now occur before the age of 55 [3]. Despite our patient only being 28 years of age, his background was significant for multiple modifiable risk factors, including hypertension, type 2 diabetes mellitus, dyslipidaemia, obesity, and smoking. Conventional risk factors have been found to be strong contributors in young stroke alike, with 10 modifiable risk factors (Figure 7) accounting for a combined population-attributable risk of 92.2% for stroke in people under the age of 55 [8]. This highlights the importance of early recognition and modification of such risk factors in both prevention and management of vascular events.

**Figure 7:**
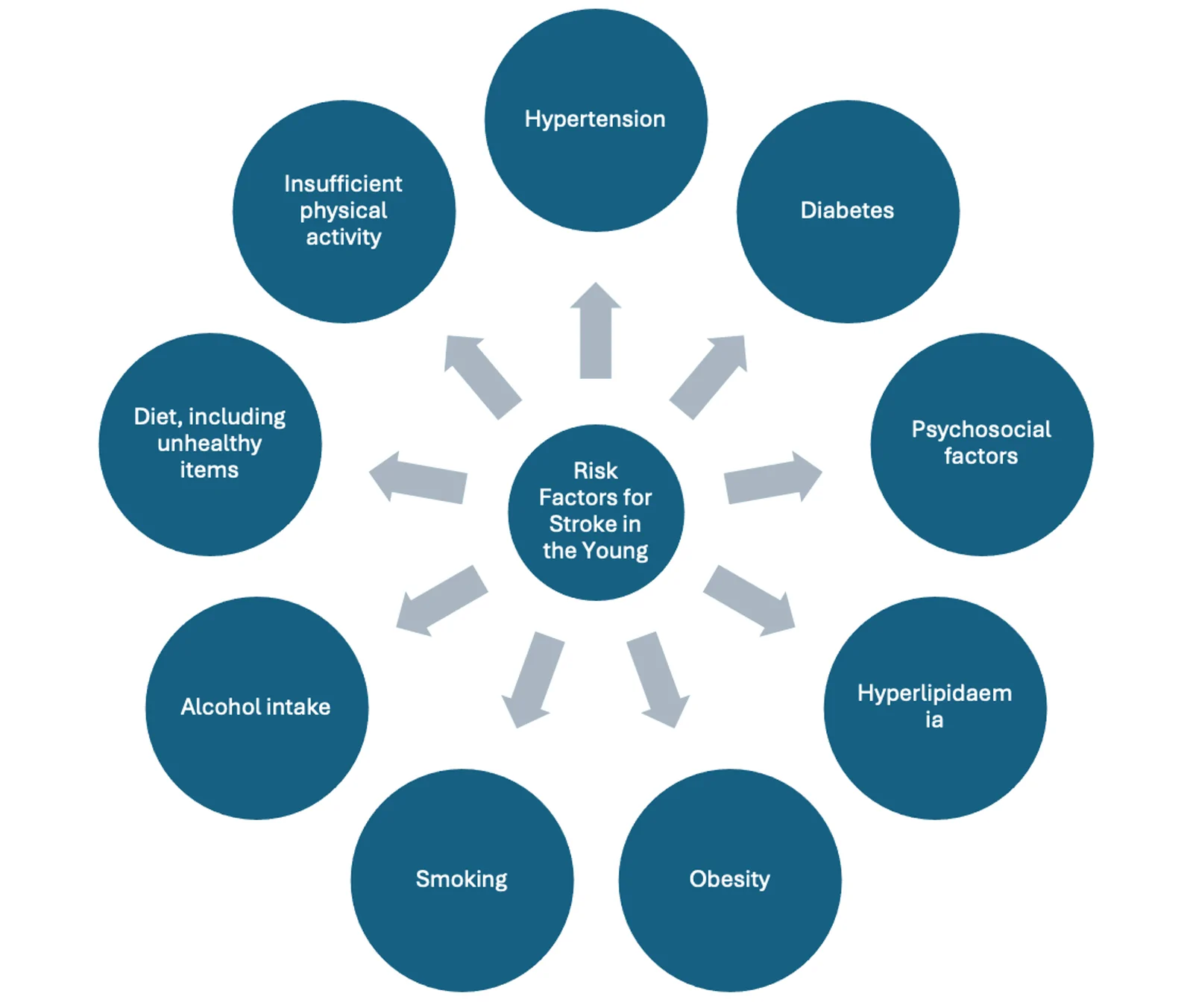
Modifiable risk factors for stroke in the young

Following initial management in our patient for a conventional ischaemic stroke, residual changes within the splenium of the corpus callosum of the right cerebral hemisphere, which did not show progression or regression between studies, raised suspicion of a possible CLOCC. A systematic review of CLOCCs found that the most affected region of the corpus callosum is within the splenium, and restricted diffusion occurs in >99% of CLOCCs [9]. Our patient’s lesion shared both characteristics. A review by Moors et al. also concluded that CLOCCs were generally reversible, commonly within a few weeks [9]. In this case, serial MRI showed near-complete regression, further supporting CLOCC as a possible diagnosis.

Many triggers have been identified for CLOCCs, including metabolic causes. Our patient had a background of poorly controlled type 2 diabetes mellitus, with an HbA1c of 109 mmol/mol at presentation. This markedly raised HbA1c increased suspicion of a metabolic cause of CLOCC in our patient. A case of CLOCC in a 28-year-old female published by Scoppettuolo et al. was also associated with hyperglycaemia [10]. Their patient had significant ketotic hyperglycaemia (blood glucose 352 mg/dL), with newly diagnosed type 1 diabetes mellitus. Contrastingly, our patient had established type 2 diabetes mellitus and chronic dysglycaemia. Interestingly, both cases demonstrate similar imaging findings of splenial lesions with diffusion abnormalities. Both cases demonstrated radiological regression, although contrastingly, the lesion reported by Scoppettuolo et al. resolved completely by 12 weeks, whilst MRI brain in our patient showed the lesion was still visible at 14 weeks. An eventual reduction in HbA1c to 63 mmol/mol correlated with near-complete regression of the splenial lesion in our patient. Improvements in glycaemic control occurring alongside lesion regression support the hypothesis of a possible metabolic contribution in this case, and hyperglycaemia-induced inflammatory/cytokine dysregulation may be responsible [10].

Although a CLOCC is a plausible explanation for the splenial lesion in this patient, alternate pathologies must be considered. One important alternative diagnosis is a splenial infarction, with the initial MRI brain characterising the splenial lesion as an acute infarction. The blood supply of the corpus callosum is from both the anterior and posterior circulation, with the posterior pericallosal artery, a branch of the PCA, supplying the splenium [11]. CTA in our patient revealed a right PCA occlusion. This shows an anatomical mechanism that supports a right splenial infarct. The splenium has also been found to be the most susceptible region of the corpus callosum to infarction [11], further supporting this hypothesis and concluding that location alone does not distinguish CLOCC from infarction. It has also been found that callosal infarcts are usually associated with other ischaemic lesions [11], and in this case, the patient had a co-existing acute right hippocampal infarction. Despite such evidence supporting a vascular explanation, the lesion did not behave like a typical infarct, and the eventual near-complete regression increases suspicion of an alternative process. There is therefore an argument for both interpretations; PCA occlusion with vascular anatomy and concurrent infarct within the hippocampus favour ischaemia, whilst the atypical course and eventual near-complete regression favour the possibility of CLOCC.

Splenial-restricted diffusion lesions can create a diagnostic challenge. In our case, there is evidence to support vascular pathology, given the confirmed right PCA occlusion and right hippocampal infarct. The initial management of this as an infarction was therefore not unreasonable. Subsequent serial imaging was crucial in this case, as the splenial lesion did not evolve as expected for a conventional infarct. This prompted reconsideration of the initial diagnosis. MRI showing subsequent near-complete regression further supported the possibility of an alternative or concurrent process. Interpretation and subsequent management should incorporate lesion location, vascular anatomy, associated cerebral lesions, metabolic context, and clinical context. The presence of ischaemic stroke does not necessarily indicate that other radiological abnormalities share the same underlying pathology. In our patient, the right PCA occlusion and hippocampal infarction support a definite vascular process, whilst the atypical nature of evolution and near-complete regression raise the possibility of a concurrent cytotoxic process. This highlights the importance of considering alternative or co-existing pathologies where radiological features do not fit the original diagnosis. Serial imaging may be a useful tool to assess atypical splenial lesions.

## Conclusions

Our case highlights a young patient with a confirmed ischaemic stroke and a concurrent atypical splenial lesion. CLOCC became a possible alternative or co-existing diagnosis, as subsequent radiological evolution was not entirely consistent with an infarct alone. The right PCA occlusion and hippocampal infarct collectively favour a vascular pathology. On the other hand, the eventual near-complete regression supported consideration of CLOCC, with poor glycaemic control in our patient providing a plausible trigger. Overall, CLOCC should be considered in the differential diagnosis of atypical splenial lesions. Imaging findings should be interpreted alongside vascular, metabolic and clinical context, and serial imaging may be valuable when the lesion behaves differently than expected.
